# Genotypic determination of resistance and heteroresistance to clarithromycin in *Helicobacter pylori* isolates from antrum and corpus of Colombian symptomatic patients

**DOI:** 10.1186/s12879-019-4178-x

**Published:** 2019-06-21

**Authors:** Betsy Verónica Arévalo-Jaimes, Diana F. Rojas-Rengifo, Carlos Alberto Jaramillo, Belén Mendoza de Molano, José Fernando Vera-Chamorro, María del Pilar Delgado

**Affiliations:** 1 Molecular Diagnostics and Bioinformatics Laboratory, Department of Biological Sciences, Los Andes University, Cra 1 # 18A- 10 Office J211, Zip Code, 111711 Bogotá, Colombia; 2 Gastroenterology Department, University Hospital Foundation Santa Fe de Bogotá, Bogotá, Colombia

**Keywords:** *Helicobacter pylori*, Clarithromycin, Resistance, Heteroresistance, Eradication

## Abstract

**Background:**

The effectiveness of *Helicobacter pylori* first-line treatment has decreased drastically with the rise of strains resistant to clarithromycin. Therapy failure has also been described in patients with infections by strains with dissimilar antimicrobial susceptibilities. The present study aims to estimate the prevalence of resistance and heteroresistance to clarithromycin in *H. pylori* isolates from antrum and corpus of Colombian patients.

**Methods:**

The study material included 126 isolates from antrum and corpus biopsies from 63 symptomatic patients over 18 years old who had a gastric endoscopy performed on them between June 2014 to August 2016. PCR amplification and sequencing of the *H. pylori* 23*S rDNA* gene was performed to determine the presence of mutations associated with clarithromycin resistance. Random amplified polymorphic DNA analysis was implemented in cases of resistance and heteroresistance.

**Results:**

The overall frequency of resistance to clarithromycin was 38.1% (24/63 patients), of which 19 patients had resistant isolates in both stomach segments (14 with A2143G mutation and 5 with A2142G mutation), and 5 patients had a heteroresistant status. The remaining 61.9% (39/63 patients) presented only susceptible isolates. DNA fingerprinting analysis showed different patterns in 4/22 paired isolates.

**Conclusions:**

The high prevalence of *H. pylori* clarithromycin-resistance obtained (> 15%) constitutes an alert for gastroenterologists and suggests the need for reconsideration of the current eradication regimen for *H. pylori* in the studied population. The data show that heteroresistance status is an additional factor to be considered in the assessment of resistance. In consequence, it is advisable to examine at least two biopsies from different gastric segments.

## Background

*Helicobacter pylori* infection is a public health issue worldwide. This Gram-negative bacterium is associated with diseases such as gastritis, peptic ulcer, gastric adenocarcinoma and mucosa-associated lymphoid tissue (MALT) lymphoma [1]. For that reason, it was classified as a group 1 carcinogen for stomach cancer by the International Agency for Research on Cancer [2]. *Helicobacter pylori* infection is usually acquired in childhood, and it can persist for the host’s lifetime unless being specifically treated [1]. As a result, more than 50% of the world’s population has *H. pylori* in their upper gastrointestinal tract, making it the most widespread infection in the world [1].

The first line of treatment for *H. pylori* infection is known as triple therapy because it includes a proton pump inhibitor (PPI) and two antibiotics (clarithromycin with either amoxicillin or metronidazole) [1]. However, the efficacy of this regimen has been drastically declining mainly due to an increase of *H. pylori* strains resistant to clarithromycin [3]. A previous report found that 66% of patients who had been treated unsuccessfully had clarithromycin resistant strains [4]. Resistance to clarithromycin is attributable to point mutations within the peptidyl-transferase encoding region of the *23S rDNA* gene [5]. The A2143G, A2142G and A2142C mutations block the clarithromycin binding site at the 50S bacterial ribosomal subunit, which inhibits the bacteriostatic activity of this antibiotic [5].

Moreover, *H. pylori* infection by strains with dissimilar antimicrobial susceptibilities could affect the therapy’s success [3]. This co-existence of susceptible and resistant strains to the same antibiotic in the same patient is known as heteroresistance [3]. Heteroresistant status can be developed from a pre-existing strain or may represent a mixed infection [3]. Therefore, detection of heteroresistance cases is necessary in order to not underestimate clarithromycin resistance. This makes the study of biopsies from different stomach segments advisable [6]. As a result, the possibility of detecting *H. pylori* resistant strains will increase and likewise, the probability of prescribing the appropriate treatment for the patient will also increase.

In Colombia, the selected *H. pylori* treatment is empirical standard triple therapy due to the impossibility of performing susceptibility testing in all patients. Consequently, local susceptibility patterns and studies to determine the local prevalence of antibiotic resistance could become essential to assist clinicians in selecting the most appropriate first-line treatment for their practice [7]. The prevalence of *H. pylori* resistant to clarithromycin assessed mainly through antimicrobial susceptibility methods in Colombia ranges between 2 and 20% [8]. However, heteroresistance in patients has only been reported in one previous study [9].

*23S rDNA* PCR-targeting allows the detection of *H. pylori* infection but may also provide information about antimicrobial susceptibility via DNA sequencing of PCR products [10]. The present study aims to estimate the prevalence of resistance and heteroresistance to clarithromycin in symptomatic Colombian adult patients through amplification and sequencing of the *23S rDNA* gene of *H. pylori* isolates from stomach antrum and corpus. In this way, it intends to offer information that may help gastroenterologists to improve *H. pylori* treatment in the studied population.

## Methods

### Samples

The Molecular Diagnostics and Bioinformatics Laboratory from Los Andes University in Bogotá-Colombia evaluated the antrum and corpus of 340 adult patients (over 18 years old) and created a bank of strains with the positive isolates for *H. pylori* growth. Individuals with digestive symptoms indicating the need for an upper digestive endoscopy had the procedure done at the University Hospital Foundation Santa Fe de Bogotá, Colombia, between June 2014 to August 2016. The inclusion criteria were upper abdominal pain, dysphagia, dyspepsia, retrosternal pain, chronic diarrhea, persistent nausea, gastroesophageal reflux, and gastrointestinal bleeding. The exclusion criteria were coagulopathy, amyloidosis, cardiovascular disease, and respiratory disease. Patients with cancer that had been exposed to chemotherapy and radiotherapy 6 months earlier were also excluded as well as patients who had ingested antacid 12 h before the procedure, PPIs/H2 blockers 15 days earlier, or antibiotics the previous month.

Information about demographic and socioeconomic factors, family medical history, and personal medical records of enrolled patients was previously collected by a questionnaire. Furthermore, we had results for the rapid urease test (RUT), which was conducted from an additional antrum biopsy with the Sensibacter pylori-Test® (Laboratorio Microanálisis Ltda, Bogotá, Colombia) according to the manufacturer’s instructions.

**Fig. 1:**
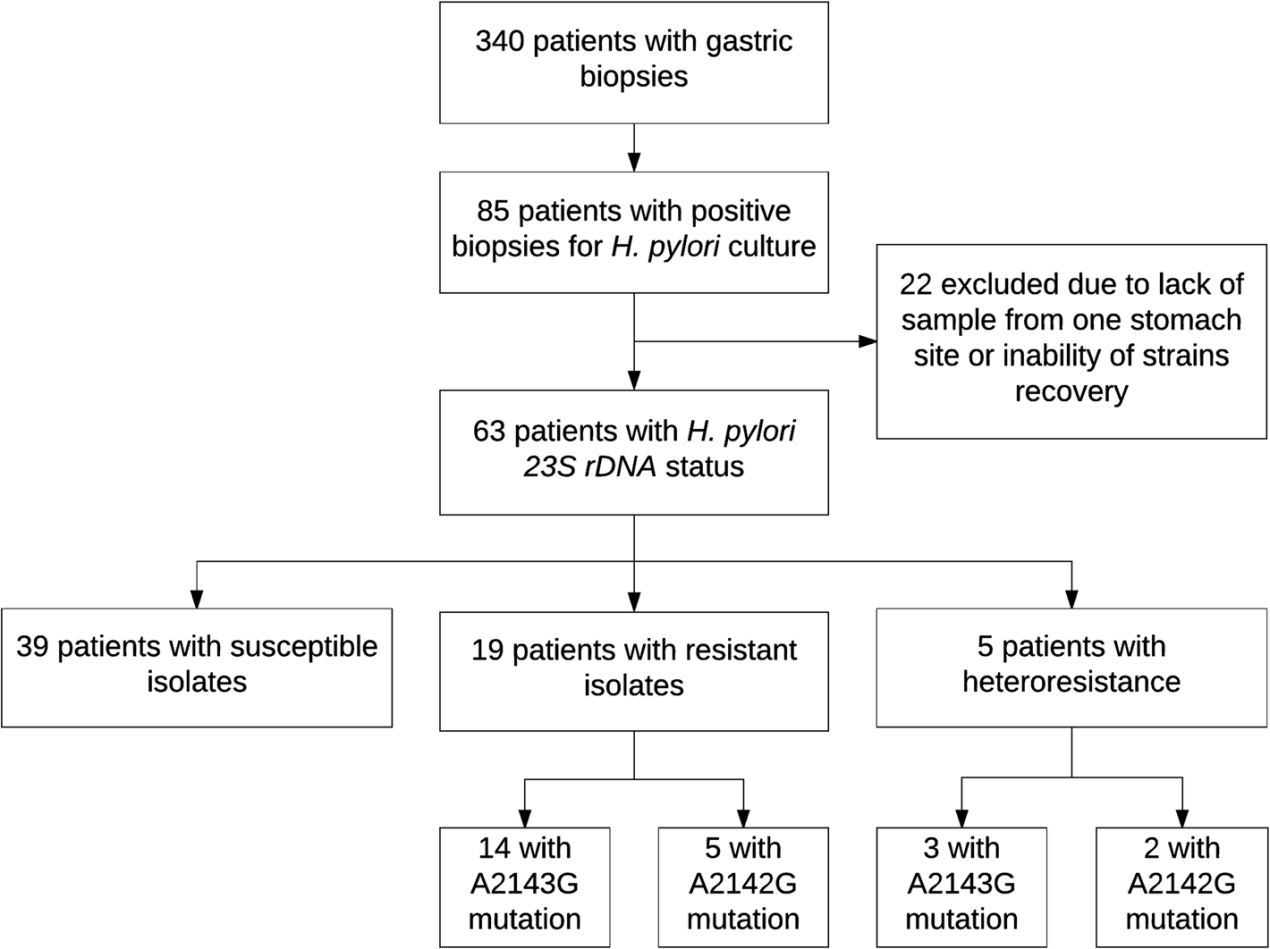
Study Profile. Flowchart of the selection process of the study sample and their distribution per *H. pylori 23S rDNA* status

A total of 85 patients were positive for *H. pylori* culture. Due to the lack of *H. pylori* growth in one of the two stomach sites, 15 patients were dismissed. Also, because of the inability of some strains to recover, 7 patients were excluded. The remaining 63 patients (126 single isolates from antrum and corpus of *H. pylori*) were included in the present study (Fig. 1). Bacteria were recovered in GC Agar plates supplemented with a cholesterol/lipid mix (Gibco, Life Technologies) and the vitamin mix described by Jimenez-Soto et al. [11] at 37 °C for 4–10 days in controlled microaerophilic conditions of 10% CO_2_.

### DNA extraction and PCR reaction

Individual colonies from the *H. pylori* cultures of each stomach site were used for DNA extraction with the Quick-gDNA Miniprep Kit (Zymo Research, CA, USA) as the manufacturer’s instructions. DNA was stored at − 20 °C until further use. Conventional PCR using primers HPYS and HPYA and cycling conditions according to Álvarez et al. [12] was performed for the amplification of a 267 bp fragment of the *23S rDNA* gene. Reactions were completed in 25 μL containing 2X GoTaq® Green Master Mix (Promega, WI, USA), 10 pmol/μL of each primer, and 2 μL of genomic DNA. The PCR products were separated in 2% (w/v) agarose gel in a TAE 0.5X (Tris/Acetate/EDTA) buffer under 80 V for 100 min. Bands were visualized with the ChemiDoc™ XRS system (Bio-Rad, CA, USA) using GelRed™ Nucleic Acid Gel Stain (Biotium, CA, USA).

### Sequencing and bioinformatics analysis

The PCR products were purified and sequenced at Macrogen.Inc. (Seoul, Korea). The sequences were edited and assembled with CLC Genomics Workbench 8 software (https://www.qiagenbioinformatics.com/). The identity of the sequences was confirmed with a BLASTn at NCBI. Chromatogram analysis was performed with CodonCode Aligner (v. 6.0.2, Codon Code Corpustion) to detect heterozygosity in the two copies of the *23S rDNA* gene. Finally, we created paired alignments with a sequence of a clarithromycin-susceptible *H. pylori* strain (GenBank accession number U27270). The positions of the mutations within the bacteria genome were determined according to the method previously described [5].

### Random amplified polymorphic DNA (RAPD)–PCR amplification

To analyze heteroresistance and resistance cases, the genotypes of paired isolates (antrum and corpus) were compared using a random amplified polymorphic DNA (RAPD)-PCR procedure. RAPD-PCR was carried out in 15 μL volume with 20 pmol of primer, 2X of GoTaq® Green Master Mix and 1 μL of genomic DNA. The primer D9355 (5′-CCGGATCCGTGATGCGGTGCG-3′) was used [13]. The thermic profile was modified from Akopyanz et al. [13]. The adapted amplification consisted of 5 low stringency cycles followed by 30 high stringency cycles. The initial 5 cycles were touchdown: 94 °C (5 min), 40 °C–35 °C (5 min) and, 72 °C (5 min); the initial annealing temperature of 40 °C was reduced 1 °C after each cycle. The following 30 cycles consisted of 94 °C (1 min), 55 °C (1 min), and 72 °C (2 min). A final extension step was performed at 72 °C for 10 min. The analysis of RAPD-PCR patterns was performed by electrophoresis in a 2% (w/v) agarose gel under 50 V for 80 min.

RAPD-PCR amplification was performed by triplicate to get reproducible results. To compare the DNA fingerprinting patterns of the paired isolates, we performed an analysis with ImageJ 1.51j8 software (National Institute of Health, USA). The criteria used for the classification of the fingerprint patterns were the same used by Selgrad et al., which is as follows: (1) identical when fingerprint patterns were equal; (2) similar when fingerprints had the same patterns with additional or different sized bands; or (3) different when the fingerprint patterns were distinct [6].

### Statistical analysis

Associations of family medical history and personal medical records with antibiotic susceptibility were evaluated by the creation of a logistic regression model with a backward stepwise selection. In addition, to estimate the recovery rate of the *H. pylori* culture, the concordance between the RUT and the culture results for *H. pylori* infection of 291 patients from the initial population was calculated by Cohen’s Kappa coefficient. Statistical analyses were performed using RStudio version 0.99.467 and R version 3.2.5. A probability (p) value less than 0.05 was considered significant.

## Results

The study population consisted of 63 patients (37 females and 26 males; mean age of 50 ± 16.4 years). Most individuals had a high socioeconomic level (77.7%), an undergraduate or graduate education level (85.7%) and shared a household with 2 or 3 people (69.9%). The socioeconomic level of each person was defined as stated by the Colombian stratification system in the following way: the social stratum 1, 2, and 3 were related to a low socioeconomic level, stratum 4 was classified as a medium socioeconomic level, and stratum 5 and 6 belonged to a high socioeconomic level [14]. Detailed demographic characteristics of patients are presented in Table 1.

**Table 1:** Patient’s demographic characteristics by genotype of *H. pylori* infection

Social factors	Clarithromycin Susceptible n (%)	Clarithromycin Resistant n (%)	Total n (%)
Sex			
Male	18 (46.1)	8 (33.3)	26 (41.3)
Female	21 (53.9)	16 (66.7)	37 (58.7)
Age (years)			
18 a 30	8 (20.5)	2 (8.3)	10 (15.9)
31 a 40	3 (7.7)	4 (16.7)	7 (11.1)
41 a 50	9 (23.1)	5 (20.8)	14 (22.2)
51 a 60	11 (28.2)	7 (29.2)	18 (28.6)
61 a 70	5 (12.8)	4 (16.7)	9 (14.3)
> 71	3 (7.7)	2 (8.3)	5 (7.9)
Socioeconomic Level			
Low	2 (5.1)	–	2 (3.2)
Medium	12 (30.8)	–	12 (19)
High	25 (64.1)	24 (100)	49 (77.8)
Education Level			
School	2 (5.1)	–	2 (3.2)
High school	3 (7.7)	2 (8.3)	5 (7.9)
Technician	2 (5.1)	–	2 (3.2)
Undergraduate	28 (71.8)	15 (62.5)	43 (68.3)
Graduate	4 (10.3)	7 (29.2)	11 (17.5)
No. of people in the household			
≤ 1	3 (7.7)	4 (16.7)	7 (11.1)
2	9 (23.1)	8 (33.3)	17 (27)
3	21 (53.8)	6 (25)	27 (42.9)
≥ 4	6 (15.4)	6 (25)	13 (20.6)
Total	39	24	63

**Fig. 2:**
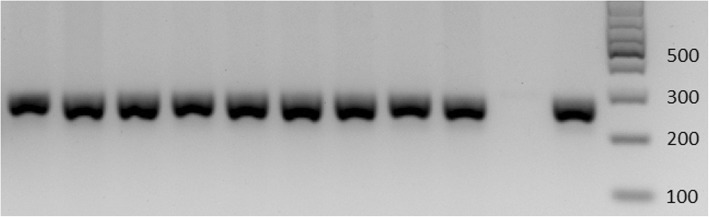
PCR products for a 267 bp region of the *23S rDNA* gene of *Helicobacter pylori*. 2% (w/v) Agarose gel. Line 1–9: Bacterial samples. Line 10: Blank of reaction. Line 11: Positive control *Helicobacter pylori* NCTC 11637. Line 12: 100 bp Leader

All 126 isolates from the antrum and corpus of 63 patients showed the expected band (267 bp) after going through the PCR protocol for the amplification of the *23S rDNA* gene (Fig. 2). The sequences obtained after edition and assembling were deposited in the GenBank database (accession numbers KY694038-KY694163). Besides, paired alignments with the reference sequence U27270 enabled us to classify the isolates as resistant or susceptible according to the presence or absence of mutations (Table 2). The overall prevalence of *H. pylori* isolates resistant to clarithromycin is 38.1% (24/63 patients), of which 19 patients had a resistant isolate in both stomach fragments while 5 patients had a heteroresistant status. Detailed information about the mutations found can be observed in Fig. 1 which shows that double mutations were not found. Likewise, the information regarding the mutations’ distribution by stomach location in heteroresistance cases is presented in Table 3. These results already evaluated the presence of heterozygosity in the *23S rDNA* gene. Both the wild-type and mutated *23S rDNA* gene copies were found in three isolates. Two of these belonged to patients with a heteroresistant status (Patient 62 and 172).

**Table 2:** The genotype of *H. pylori* infection in 63 patients

Infection Genotype	n (%)
Antrum-Corpus	
Susceptible-Susceptible	39 (61,90)
Resistant-Resistant	19 (30,16)
Susceptible-Resistant	3 (4,76)
Resistant-Susceptible	2 (3,17)
Total	63 (100)

**Table 3:** *Helicobacter pylori* genotype in the heteroresistant cases by stomach location

Patient	Stomach Location	
	Antrum	Corpus
62	Wild Type	A2142G
66	A2142G	Wild Type
172	Wild Type	A2143G
243	Wild Type	A2143G
293	A2143G	Wild Type

**Fig. 3:**
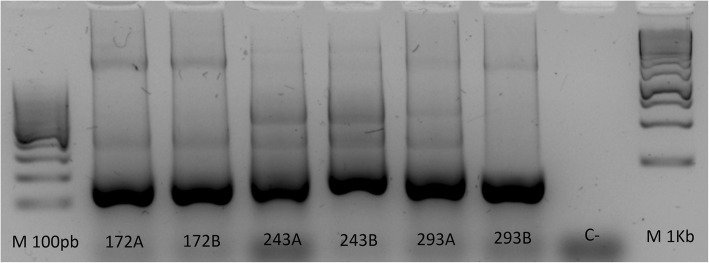
RAPD-PCR fingerprinting patterns of *Helicobacter pylori* isolates in three heteroresistance cases. It can be seen when the isolate belonged to antrum (A) or corpus (B). M: Molecular size marker. C-: Blank of reaction. Different patterns are only observed in patient 293

**Fig. 4:**
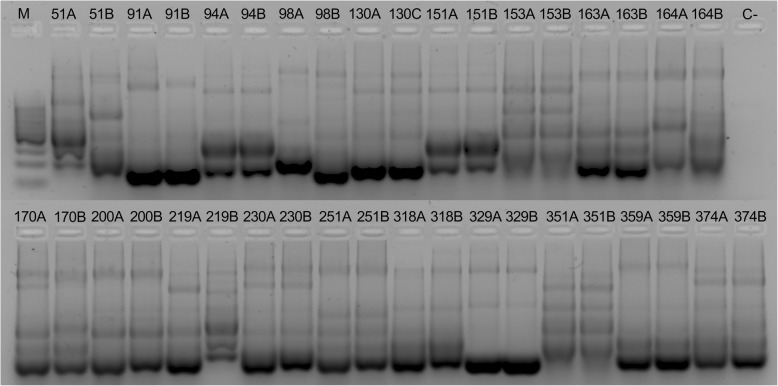
RAPD-PCR fingerprinting patterns of *Helicobacter pylori* in resistance cases. It can be seen when the isolate belonged to antrum (A) or corpus (B). M: Molecular size marker. C-: Blank of reaction. Patients 94, 130, 151, 153, 163, 200, 219, 230, 251, 318, 329, 351, 359 and 374 show identical DNA profiles. Patients 91 and 170 show similar patterns. Patients 51, 98 and 164 show different DNA profiles

The fingerprinting DNA analysis of heteroresistance cases shows two paired isolates with identical patterns and one with different patterns (Fig. 3). Samples of the remaining two heteroresistance cases were not available to conduct the RAPDs experiments due to the inability of the strains to recover. About the RAPDs banding profiles of paired isolates with clarithromycin resistance, 14 show identical patterns while 2 reveal similar patterns, and 3 demonstrate different patterns (Fig. 4).

The final logistic regression model includes variables of previous use of antimicrobials, previous therapy for *H. pylori* eradication, and the time from the onset of symptoms. This model explains the 13.8% of the cases with a mutation associated with *H. pylori* clarithromycin resistance. It was found that the risk of having a resistant strain increase 3.45-fold if the patient was subjected to a previous eradication therapy for *H. pylori* infection (CI 95% = 1.09–11.46, *p*-value = 0.037). Regarding the other statistical analysis, the concordance of the RUT and the culture for the definition of *H. pylori* infection was strong [15] (Kappa coefficient = 0.87, p-value = 1.3E-151 and CI 95% = 0.81–0.94).

## Discussion

Although most studies of *H. pylori* clarithromycin resistance in Colombia have reported prevalence equal to or lower than 20% [8], a higher percentage of 38.1 was found in our sample. A similar result (39.2%) can be found in a contemporary study completed at the Clínica Fundadores in Bogotá [16]. Also, the most recent multicenter studies conducted in developed countries show an overall prevalence of clarithromycin resistance of 32.3% (23.1–45.8%) [17] in the USA and numbers ranging from 20 to 36.6% at several European countries [18]. These findings suggest that Colombia is experiencing an increasing trend in resistant bacteria over the years as is happening worldwide.

To discard the possibility of an overestimation of clarithromycin resistance due to a poor recovery rate at the bacterial culture stage, we calculated the concordance between the RUT and the culture to define the *H. pylori* infection of 291 patients that belonged to the initial population (*n* = 340). It is known that *H. pylori* bacteria in a viable but non-culturable (VBNC) state maintain detectable levels of urease activity [19]. The outcome of Cohen’s Kappa coefficient (0.87) shows a strong concordance between the two evaluated methods which indicates that *H. pylori* bacteria detected from biopsy tissue was mostly recovered (66/76 strains, data not shown) at the culture stage.

Local variation in *H. pylori* antibiotic resistance can be explained mainly by differences in the method used to assess the susceptibility and the level of exposition to macrolides of the population. Though conventional methods for assessing resistance are antimicrobial susceptibility testing, this study implemented molecular procedures. This alternative is possible due to the small number of mutations in the *23S rDNA* gene implicated in macrolide resistance [20] and the high degree of association between phenotypic and genotypic detection of clarithromycin resistance previously found in Colombian *H. pylori* strains [9, 20]. However, genotypic resistance can detect the heteroresistant status [9], making molecular techniques more reliable than sensitivity tests in these cases, and possibly explaining the higher prevalence found.

The obtained data reaffirms a current high rate of genotypic prevalence of *H. pylori* resistant to clarithromycin in Bogotá, as Trespalacios et al. reported [16]. The correlation between genotypic resistance and therapeutic outcomes after clarithromycin-based triple therapy shows an eradication success of 21 and 86.8% in isolates with and without *23S rDNA* mutations, respectively [21]. In addition, this study shows that most resistant cases harbor the A2143G mutation (14/19 equivalent to 22% of the sample) which has been directly related to a decrease in the eradication therapy success [21]. Consequently, the eradication regimen for *H. pylori* prescribed for symptomatic adults that attend the University Hospital Foundation Santa Fe de Bogotá should be reconsidered. According to the V Maastricht/Florence Consensus Conference, clarithromycin-containing triple therapy without prior susceptibility testing should be abandoned when the clarithromycin resistance rate in the region is over 15% [22]. Consistent with the recommendations, the Clinical Practice Guidelines for the Diagnosis and Management of Adult Patients with *Helicobacter pylori* Infection suggests the bismuth-containing quadruple therapies for first-line empirical treatment in Colombia [23].

Gastric biopsy sampling could affect the accuracy of assessing *H. pylori* clarithromycin resistance due to the presence of heteroresistance. This study found a discordant susceptibility genotype between the evaluated stomach sites in 5/63 patients (7.9%), which can be contrasted with the previous record of 4/256 patients (1.6%) [9]. Infections with different antibiotic susceptibility led to a failure of 16.4% of treatments, and neither the antrum nor corpus alone is a fully representative site for the detection of antibiotic-resistant *H. pylori* [4]. Therefore, biopsies from several stomach sites might increase the diagnostic yield of *H. pylori* detection, especially due to the patchy distribution of the bacteria [6]. For practical and economical purposes, biopsies can be analyzed together [6].

DNA fingerprinting analysis of heteroresistant and resistant cases (Fig. 3 and Fig. 4) shows that the majority of patients have an infection with a single strain in both stomach segments, except for four that display patterns that support a mixed infection. The emergence of antibiotic resistance in vivo could be the result of the combined effects of the spontaneous mutation rate and the recombination mechanisms of *H. pylori* [5, 24]. Furthermore, the use of antimicrobials or a previous *H. pylori* eradication regimen could have acted as a selection pressure and favored the presence of resistant isolates, as found by the logistic regression model performed (OR = 3.45).

An important consideration of the study limitations is the proposal of novel mechanisms of resistance acquisition to clarithromycin in *H. pylori* [25]. Despite point mutations in the *23S rDNA* gene remaining the main cause, studies are needed to elucidate the implications of the discoveries that have been made and to re-evaluate whether molecular analysis limited to this gene could result in the underestimation of resistance to clarithromycin. Likewise, molecular techniques in gastric tissue should be employed to guarantee the detection of all *H. pylori* forms because VBNC forms may remain latent for a long time and contribute to treatment failures and recurrence [19]. However, this study performs molecular methods in cultures from a single colony of each gastric sample in order to not misclassify intra-niche heteroresistant as heterozygosity of *23S rDNA* gene copies and vice versa. Finally, it should be noted that no discrimination was made between primary or secondary resistance because the medical history data were obtained through a questionnaire, which does not guarantee sufficiently reliable data. Nonetheless, the calculated values were 29.3% (12/41) of primary resistance and 54.5% (12/22) of secondary resistance, which are numbers that still overcome the upper limit (15%) for the prescription of clarithromycin-triple standard therapy as the empirical first line of treatment.

## Conclusions

In conclusion, the prevalence of *H. pylori* resistant to clarithromycin found in this study suggests the need to re-evaluate the therapy in the studied population. New antimicrobial resistance studies should be conducted periodically and regionally in our country to provide information that may help to implement more effective eradication programs. Moreover, the presence of heteroresistant cases strongly recommends that prevalence studies and individual susceptibility tests should be completed by sampling biopsies from at least two different stomach locations. In this way, the data obtained will be a better representation of the actual situation of patients and populations. Finally, it is important to highlight the usefulness of molecular methods like PCR-sequencing for the detection and characterization of *H. pylori* infection as an alternative to conventional antimicrobial tests, especially to identify heteroresistant cases.

## Data Availability

The sequences generated and analyzed during the current study are available in the GenBank repository, [https://www.ncbi.nlm.nih.gov/popset?DbFrom=nuccore&Cmd=Link&LinkName=nuccore_popset&IdsFromResult=1343307132].

## Abbreviations

MALT: Mucosa-associated lymphoid tissue
PPI: Proton pump Inhibitor
RAPD: Random amplified polymorphic DNA
RUT: Rapid urease test
VBNC: Viable but non-culturable
